# Poly(A) capture full length cDNA sequencing improves the accuracy and detection ability of transcript quantification and alternative splicing events

**DOI:** 10.1038/s41598-022-14902-7

**Published:** 2022-06-22

**Authors:** Hiroki Ura, Sumihito Togi, Yo Niida

**Affiliations:** 1 Center for Clinical Genomics, Kanazawa Medical University Hospital, 1-1 Daigaku, Uchinada, Kahoku, Ishikawa 920-0923 Japan; 2 Division of Genomic Medicine, Department of Advanced Medicine, Medical Research Institute, Kanazawa Medical University, 1-1 Daigaku, Uchinada, Kahoku, Ishikawa 920-0923 Japan

**Keywords:** Molecular medicine, Clinical genetics

## Abstract

The full-length double-strand cDNA sequencing, one of the RNA-Seq methods, is a powerful method used to investigate the transcriptome status of a gene of interest, such as its transcription level and alternative splicing variants. Furthermore, full-length double-strand cDNA sequencing has the advantage that it can create a library from a small amount of sample and the library can be applied to long-read sequencers in addition to short-read sequencers. Nevertheless, one of our previous studies indicated that the full-length double-strand cDNA sequencing yields non-specific genomic DNA amplification, affecting transcriptome analysis, such as transcript quantification and alternative splicing analysis. In this study, it was confirmed that it is possible to produce the RNA-Seq library from only genomic DNA and that the full-length double-strand cDNA sequencing of genomic DNA yielded non-specific genomic DNA amplification. To avoid non-specific genomic DNA amplification, two methods were examined, which are the DNase I-treated full-length double-strand cDNA sequencing and poly(A) capture full-length double-strand cDNA sequencing. Contrary to expectations, the non-specific genomic DNA amplification was increased and the number of the detected expressing genes was reduced in DNase I-treated full-length double-strand cDNA sequencing. On the other hand, in the poly(A) capture full-length double-strand cDNA sequencing, the non-specific genomic DNA amplification was significantly reduced, accordingly the accuracy and the number of detected expressing genes and splicing events were increased. The expression pattern and percentage spliced in index of splicing events were highly correlated. Our results indicate that the poly(A) capture full-length double-strand cDNA sequencing improves transcript quantification accuracy and the detection ability of alternative splicing events. It is also expected to contribute to the determination of the significance of DNA variants to splicing events.

## Introduction

The large majority of human genes are processed at several levels, including transcriptional and post-transcriptional regulation. Alternative splicing of pre-mRNAs that include exons and introns is one of the essential regulatory mechanisms at post-transcriptional regulation^1,2^. Alternative splicing plays an important role in normal cellular and pathogenic processes caused by diverse diseases^3,4^. It has been reported that several alternative splicing events, including alternative 5’ or 3’ splicing site usage, exon skipping, intron retention, and mutually exclusive exons, occur in abnormal cells in various diseases and normal cells^5–7^. These alternative splicing events produce assorted mRNA that translates to different protein isoforms with different coding sequences. In normal cells, these alternative splicing events are controlled in an appropriate expression pattern^8–10^. Alternatively, an inappropriate expression pattern occurs in some human diseases, including cancers^11–13^. Therefore, it is important to accurately analyze the state of the repertoire of mRNA splicing variants and its changes associated with the pathological condition.

Next-generation sequencing (NGS) is a powerful technology used in the clinical field for genetic diagnosis^14–16^. The use of NGS technologies in the clinical field has led to an unprecedented increase in variants identified in different patients harboring genetic disorders. The 48% of all variants listed on ClinVar are asserted to the variant of uncertain significance (VUS) variants^17^. The current genetic counseling practice almost considers variants that directly affect protein structure. However, the VUS can affect RNA splicing, which causes protein damage. RNA splicing is expected to be disrupted by approximately 62% of all pathogenic variants^18^. It is also reported that aberrant RNA splicing affects the transcription level^19^. Thus, it is also needed to accurately measure RNA splicing and transcription level in precise genetic diagnosis.

**Figure 1:**
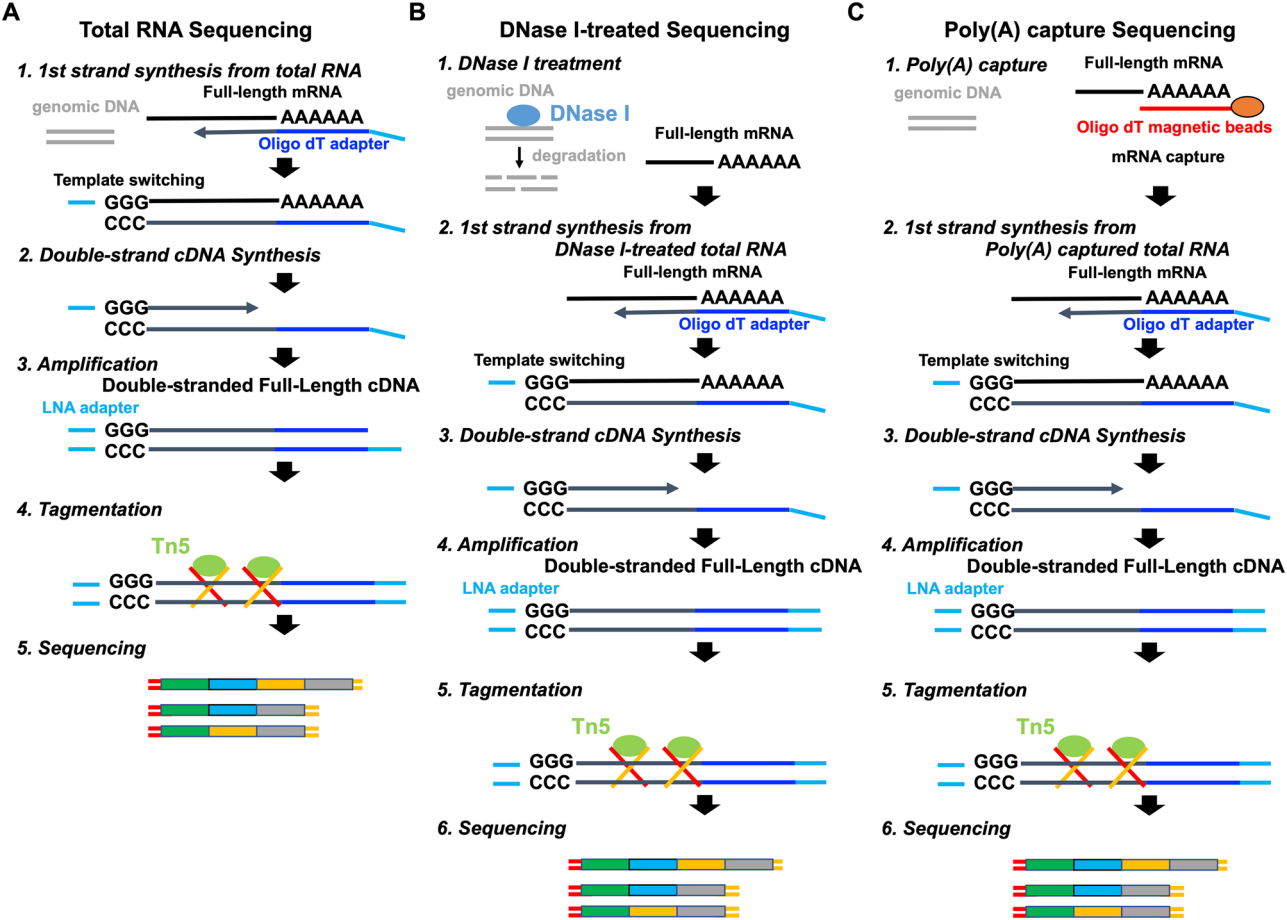
Library preparation workflow. (**A**) Workflow for Total RNA Sequencing. (**B**) Workflow for DNase I-treated Sequencing. (**C**) Workflow for Poly(A) capture Sequencing.

RNA sequencing (RNA-Seq) is a powerful technology that can be used to measure not only transcriptional levels but also alternative splicing repertoire^20^. However, so far, RNA-Seq is primarily used to measure the expression level of transcripts but rarely used to detect alternative splicing variants^21,22^. Presently, RNA-Seq is almost performed using short-read sequencers, such as the Illumina NGS. Although RNA-Seq by short-read sequencer can detect alternative splicing events between two exons, short-read RNA-Seq cannot detect the full-length transcript information, including all alternative splicing events. Recently, third-generation sequencers, such as Oxford Nanopore Technologies (ONT) and Pacific Biosciences (PacBio), can be facilitated for alternative splicing analysis of the full-length transcript due to the production of long sequencing reads (> 10 kb)^23^. When creating a library in traditional RNA-Seq, because the captured mRNAs using oligo dT magnet beads are sheared randomly into fragments, then reverse transcribed into cDNAs, it is impossible to apply the library for analysing the full-length transcripts. Alternatively, due to no fragmentation, full-length double-stranded cDNA library can adapt to long-read sequencer for analysing the full-length transcript^24^. In addition, since the full-length cDNA is yielded by PCR amplification, a library can be prepared from a small amount of sample, even as a single cell (Fig. 1A). In principle, novel splicing variants caused by the DNA variants of non-coding regions can be directly clarified, if a DNA sequencing including non-coding regions such as a whole genome sequencing and a full-length cDNA sequencing are performed at the same time. Nevertheless, one of our previous studies indicated that the full-length double-strand cDNA sequencing resulted in non-specific genomic DNA amplification, which affects precise transcriptome analysis, such as alternative splicing and transcript quantification analysis^25^. For that reason, it is needed to eliminate genomic DNA noise when creating a full-length double-stranded cDNA library for more precise mRNA analysis.

To avoid contamination of genome DNA in Total RNA, we tried two possible methods and compared their efficiencies. One method (DNase I-treated full-length double-strand cDNA sequencing) is that genome DNA in Total RNA solution is digested enzymatically using DNase I enzyme^26^ (Fig. 1B). Other method (poly(A) capture full-length double-strand cDNA sequencing) is that only messenger RNA which have Poly(A) tail are physically captured using magnetic Oligo(dT) beads^27^ (Fig. 1C). It was investigated that the performance of these two methods in transcriptome analysis, such as transcript quantification analysis and alternative splicing analysis.

## Methods

### Cell culture

The human induced pluripotent stem cell-line (hiPSC), strain 1383D6, which was provided by RIKEN BioResource Research Center were cultured on iMatrix 511 (Takara)-coated plates (0.5 ug/cm^2^) in StemFit medium (REPROCELL) at 37 °C in 5% CO_2_^28^. The cells were passaged as clump with TrypLE Select (Life Technologies) at a ratio of 1:6 every 4–5 days.

### Genomic DNA extraction

The genomic DNA sample used in this study was extracted from the whole peripheral blood using a rapid extraction method^29^. The DNA amount and optical density (A260/280 ratio) were measured using Nanodrop (Thermo Fisher Scientific, Waltham, MA, USA).

### Total RNA extraction

The total RNA was extracted from hiPSCs with TRIzol reagent (Thermo Fisher Scientific) following the manufacturer’s instructions, as described previously^30,31^. RNA concentration and purity were measured spectrophotometrically (Nanodrop, Thermo Fisher Scientific). The RNA integrity number was determined using a TapeStation 4200 with High Sensitivity RNA ScreenTape (Agilent Technologies, Santa Clara, CA, USA).

### Total RNA library synthesis

Total RNA Library was synthesized from total RNA using a SMART-Seq HT kit (Takara Bio USA, Mountain View, CA, USA) and the Nextera XT DNA Library Kit (Illumina, San Diego, CA, USA), following the manufacturer’s standard protocol. The library quality was further evaluated using the TapeStation 4200 with High Sensitivity D1000 ScreenTape (Agilent Technologies, Santa Clara, CA). The library was quantified using the HS Qubit dsDNA assay (Thermo Fisher Scientific, Waltham, MA, USA).

### Genomic DNA library synthesis

Genomic DNA Library was synthesized from genomic DNA using a SMART-Seq HT kit (Takara Bio USA, Mountain View, CA, USA) and the Nextera XT DNA Library Kit (Illumina, San Diego, CA, USA), following the manufacturer’s standard protocol. The library quality was further evaluated using the TapeStation 4200 with High Sensitivity D1000 ScreenTape (Agilent Technologies, Santa Clara, CA). The library was quantified using the HS Qubit dsDNA assay (Thermo Fisher Scientific, Waltham, MA, USA).

### DNase I-treated full-length double-stranded cDNA

According to the manufacturer’s instruction, the genomic DNA contained in total RNA was digested using TURBO DNA-free Kit (Thermo Fisher Scientific). The RNA integrity number of DNase I-treated total RNA was determined using a TapeStation 4200 with High Sensitivity RNA ScreenTape (Agilent Technologies, Santa Clara, CA, USA). According to the manufacturer’s standard protocol, full-length double-stranded cDNA was synthesized from DNase I-treated total RNA using a SMART-Seq HT kit (Takara Bio USA, Mountain View, CA, USA).

### Poly(A) capture full-length double-stranded cDNA

According to the manufacturer’s instruction, the mRNA was captured from total RNA with NEBNext Poly(A) mRNA Magnetic Isolation Module (New England BioLabs). Briefly, the mRNA was captured using NEBNext Magnetic Oligo d(T)_25_ Beads and washed. The captured mRNA was resuspended with RNA binding buffer and was denatured at 65 °C for 5 min. Then, captured mRNA was eluted at 80 °C for 2 min. According to the manufacturer’s standard protocol, full-length double-stranded cDNA was synthesized from eluted mRNA using a SMART-Seq HT kit (Takara Bio USA, Mountain View, CA, USA).

### Library preparation and next-generation sequencing

The DNase I-treated or Poly(A) capture full-length double-stranded sequencing libraries were prepared using the Nextera XT DNA Library Kit (Illumina, San Diego, CA, USA) for Illumina sequencing following the manufacturer’s instructions, as described previously^32^. The library quality was further analyzed using the TapeStation 4200 with High Sensitivity D1000 ScreenTape (Agilent Technologies, Santa Clara, CA, USA). All libraries were quantified using the HS Qubit dsDNA assay (Thermo Fisher Scientific, Waltham, MA, USA). All libraries were sequenced (2 × 75 bp) using Illumina NextSeq 500 (Illumina, San Diego, CA, USA). The FASTQ files were generated using the bcl2fastq software (Illumina, San Diego, CA, USA). The FASTQ data (GSE192928) are deposited in the Gene Expression Omnibus (GEO) (https://www.ncbi.nlm.nih.gov/geo/).

### Data analysis

The FASTQ files were sampled with the same number of reads using Seqkit (version 0.13.2)^33^. The sampled FASTQ files were aligned to the reference human genome (hg38) using HISAT2 (version 2.1.0) and SAMtools (v.1.9)^34,35^. The StringTie algorithm (v.1.3.4d) was then used to assemble RNA-Seq alignment into annotated transcripts to quantify their expression^36^. The transcript expression was normalized using the transcript per million (TPM) algorithm. For differential expression analysis, the R package (edgeR) was used^37^. The mapping rate was measured using HISAT2. For alternative splicing analysis, we used SplAdder software (v.2.4.2) and RSeQC (v.3.0.1)^38,39^.

### Ethical approval

The study was conducted according to the guidelines of the Declaration of Helsinki, and the Institutional Review Board of Kanazawa Medical University (No. G111, approved November 10, 2015) approved this study. Written informed consent was obtained by Y.N., and the ethics review board of Kanazawa Medical University approved the study design (G111).

## Results

### Confirmation of Non-specific Genomic DNA Amplification from Genomic DNA

**Figure 2:**
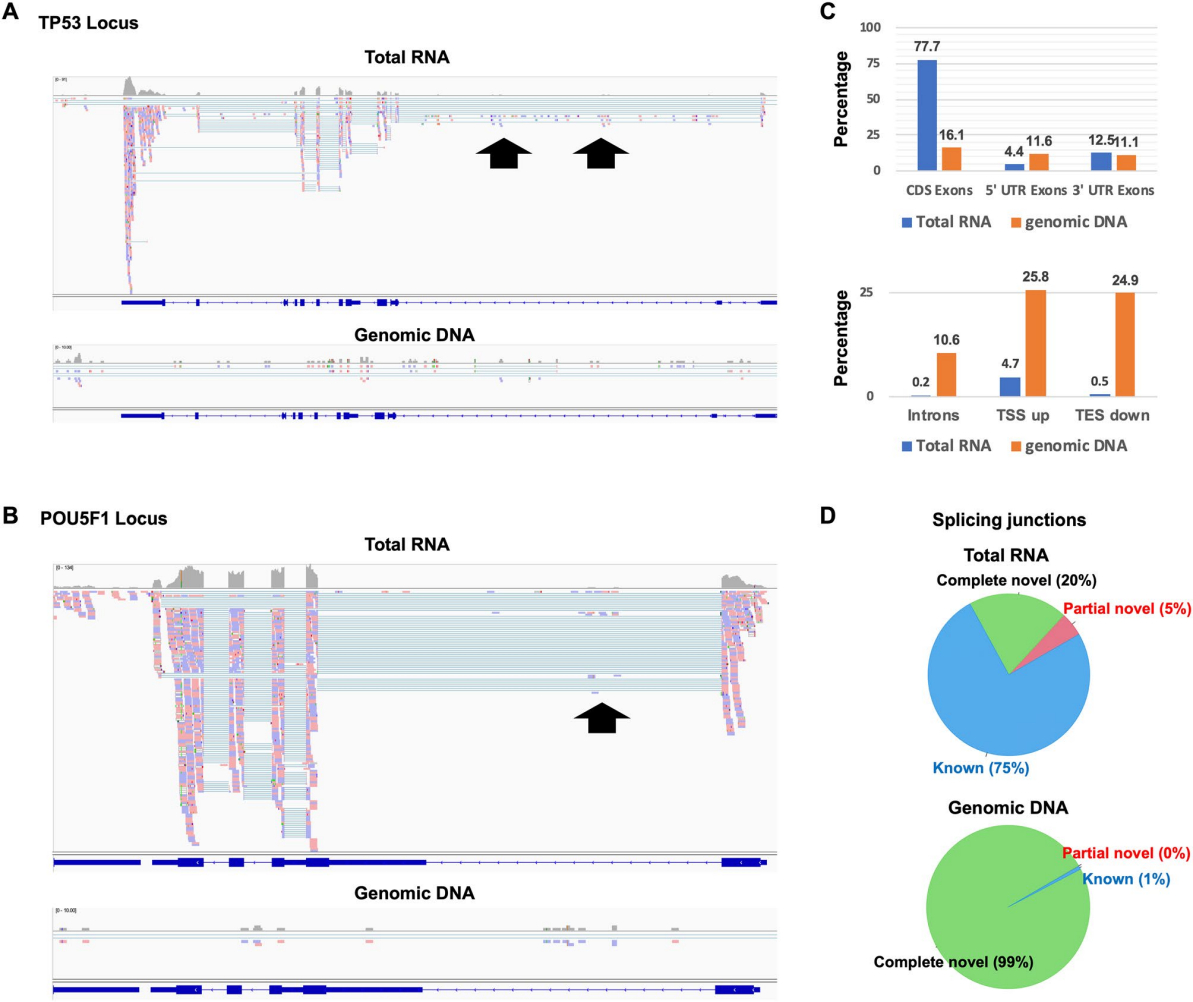
Confirmation of non-specific genomic DNA amplification from genomic DNA. (**A** and **B**) Integrative genomic viewer (IGV) of the TP53 and POU5F1 Locus mapped reads. Black arrows show non-specific genomic DNA amplification. (**C**) The percentage in each region (CDS Exons, 5’UTR Exons, 3’UTR Exons, Introns, TSS upstream (TSS up) and TES downstream (TES down)). (**D**) Pie chart of each splicing junction. The percentage of splicing events were calculated by RSeQC.

First, the full-length double-stranded cDNA library was generated from only the genomic DNA to verify that non-specific amplifications are yielded from genomic DNA. The library was generated from only the genomic DNA and to be sequenced the same as the library was generated from total RNA. Comparing to Total RNA sequencing (original SMART-Seq library), the reads of genomic DNA sequencing were randomly mapped to the genomic areas in (Fig. 2A,B and Supplementary Table S1). Although, the mapped reads in intron were also seen in Total RNA sequencing as well as genome DNA sequencing (Figs. 1B and 2A , black arrows). Next, the distribution characteristics of mapped reads were investigated (Fig. 2C and Supplementary Table S1). The distribution of coding sequence exons (CDS Exons) was enriched in Total RNA sequencing. In Total RNA sequencing, almost all reads (94.6%) were mapped to exons (CDS, 5’UTR or 3’UTR exons). On the other hand, non-coding regions (Introns, TSS upstream and TES downstream) in genomic DNA sequencing were higher than Total RNA sequencing, indicating that non-specific amplification from genomic DNA occurred. Alternatively, the distribution of each region (CDS Exons, 5’UTR Exons, 3’UTR Exons, Introns) was almost the same in genomic DNA sequencing, indicating that non-specific amplification from genomic DNA randomly occurred and was independent of sequence. The splicing junctions in Total RNA sequencing and genomic DNA sequencing were analyzed by RSeQC to assess the effect of non-specific amplification of genome DNA for alternative splicing analysis (Fig. 2D). In genomic DNA sequencing, the junctions were almost complete novel junctions (99%), showing that non-specific amplification of genome DNA may affect the detection of alternative splicing events. Although the complete novel junction in Total RNA sequencing was not as many as genome DNA sequencing (20%), the effect of non-specific amplification of genome DNA is also possible. These results suggested that it is needed to avoid contamination of genomic DNA for alternative splicing analysis.

### Comparison between total RNA, DNase I-treated, and Poly(A) capture sequencing for splicing detection accuracy

**Figure 3:**
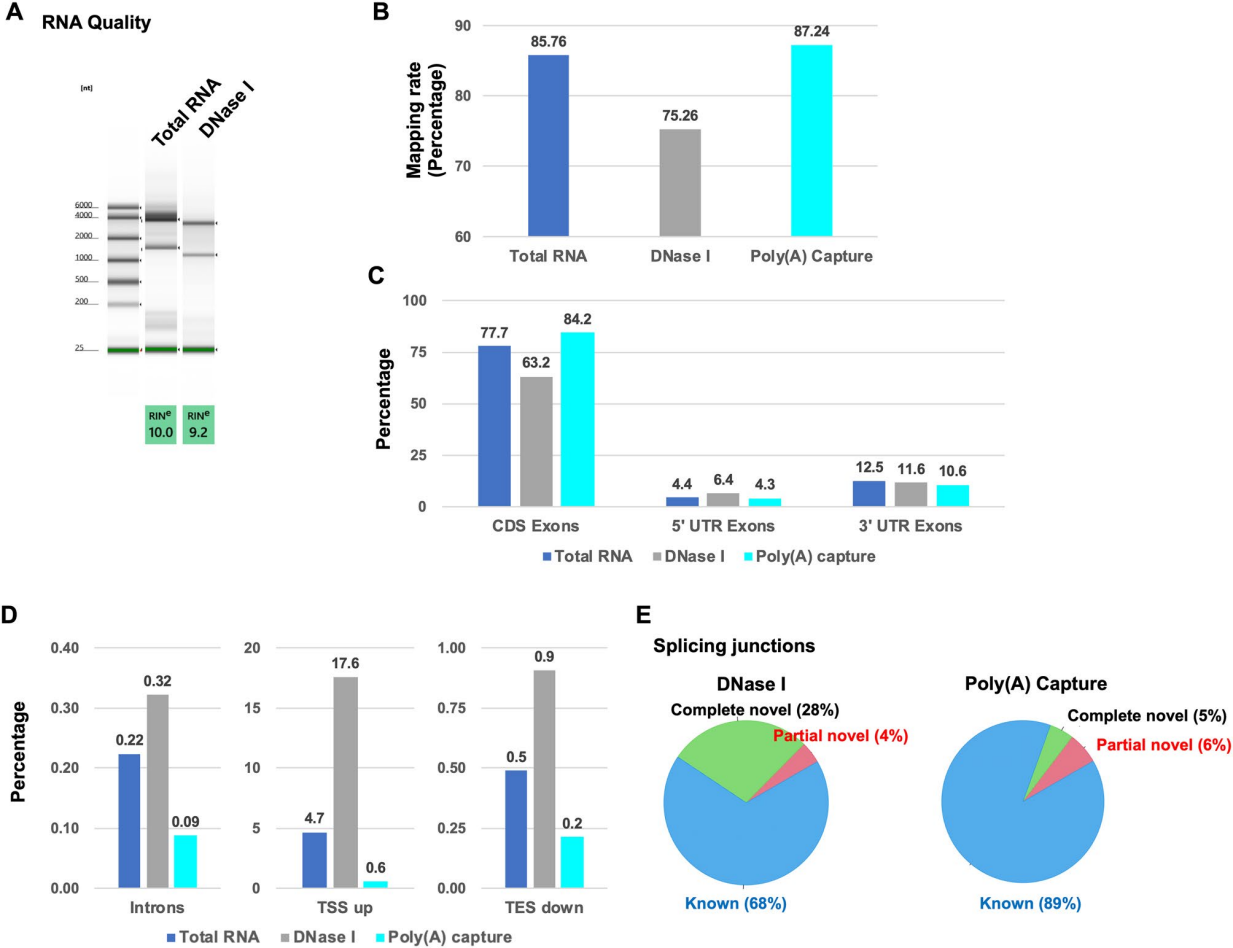
Comparison between Total RNA, DNase I-treated, and Poly(A) capture sequencing for splicing detection accuracy. (**A**) RNA quality in TapeStation. (**B**) Percentage of mapped reads. (**C**) The percentage in each region (CDS Exons, 5’UTR Exons, 3’UTR Exons). (**D**) The percentage in the genomic region (introns, TSS up, and TES down). (E) Pie chart of each splicing junction. The percentage of splicing events were calculated by RSeQC.

To evaluate the performance of Total RNA sequencing, DNase I-treated and Poly(A) capture full-length double-stranded cDNA Sequencing, their accuracy of splicing event detection was compared. The quality of DNase I-treated total RNA was the same as DNase I-untreated total RNA (Fig. 3A). The percentage of mapping rate of Poly(A) capture sequencing was slightly higher than that of Total RNA sequencing (Fig. 3B and Supplementary Table S1). In Poly(A) capture sequencing, almost all reads (99.1%) were mapped to exons. The mapping percentage of DNase I-treated sequencing was significantly lower than that of Total RNA sequencing, indicating that non-specific genomic DNA amplification in DNase I-treated sequencing affected mapping efficiency. The distribution of CDS Exons in Poly(A) capture sequencing was also slightly higher than that of Total RNA sequencing (Fig. 3C and Supplementary Table S1). The distribution of CDS Exons in DNase I-treated sequencing was lower than that in Total RNA sequencing. The distribution of 5’UTR Exons and 3’UTR Exons were almost the same between Total RNA, DNase I, and Poly(A) capture sequencing. The distribution of genomic DNA regions (introns, TSS up, and TES down) in Poly(A) capture sequencing was significantly lower than that in Total RNA sequencing (Fig. 3D). Unexpectedly, genomic DNA regions’ distribution in DNase I-treated sequencing was higher than in Total RNA sequencing. The ratio of complete novel splicing junctions of DNase I-treated sequencing (28%) was higher than that of Total RNA sequencing (20%); the ratio in Poly(A) sequencing (5%) was lower than in Total RNA sequencing (Fig. 2D, 3E). These results suggested that Poly(A) capture sequencing improves the accuracy of splicing event detection by removing contamination of genomic DNA amplification from the library, but not DNase I treatment.

### Comparison between total RNA, DNase I-treated, and Poly(A) capture sequencing for quantification analysis

**Figure 4:**
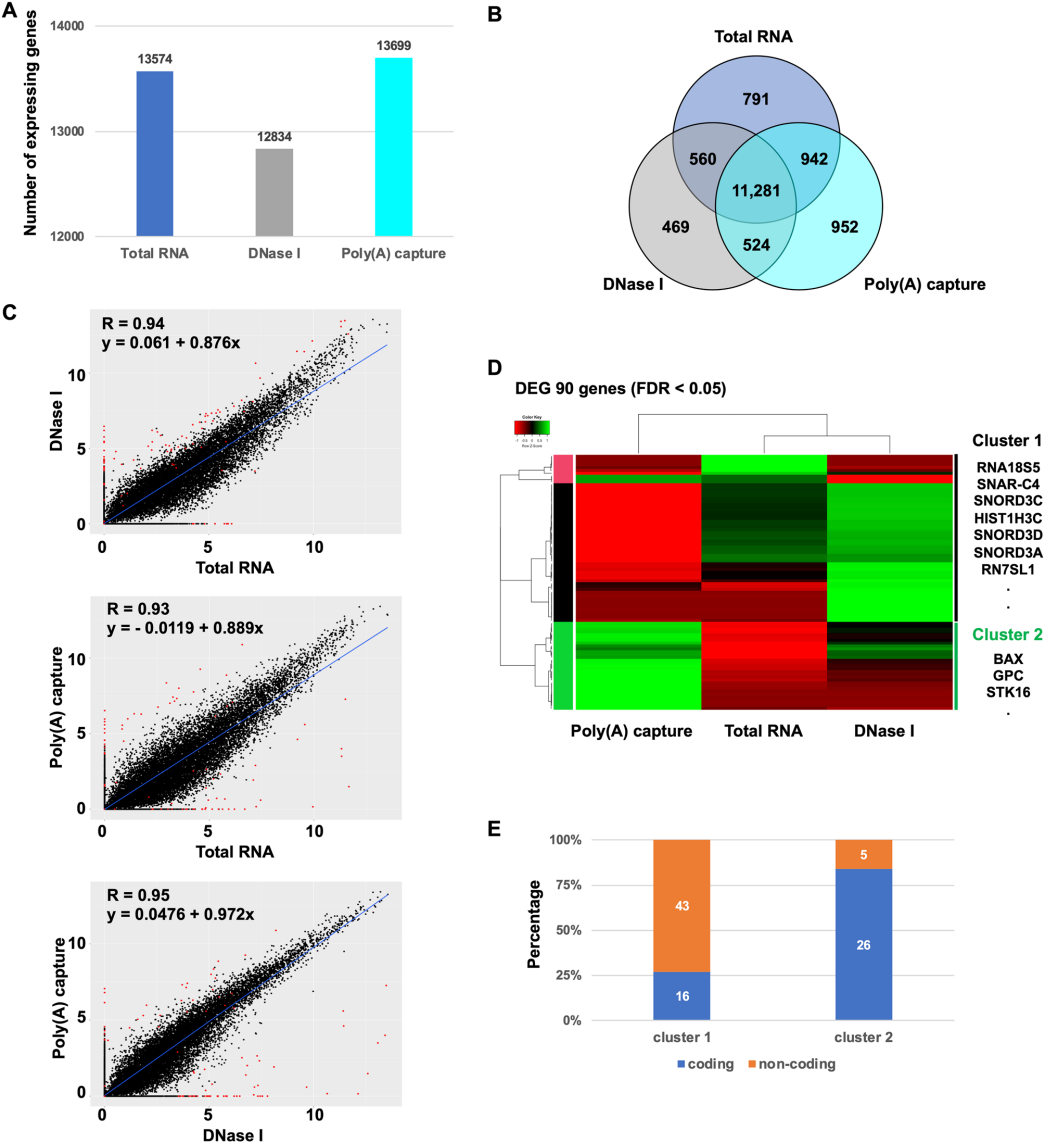
Comparison between Total RNA, DNase I-treated, and Poly(A) capture sequencing for quantification analysis. (**A**) The total number of expressed genes identified by these three methods. (**B**) Venn graph of expressed genes. (**C**) Scatter plot (log2 TPM (Transcripts per million)) of comparing each two methods. (**D**) Heat map of hierarchical clustering between total RNA, DNase I-treated, and Poly(A) capture sequencings. (**E**) The percentage of coding and non-coding genes in cluster 1 and 2.

To evaluate the performance of Total RNA, DNase I-treated, and Poly(A) capture sequencing, their accuracies of gene detection and expression patterns were compared. The number of detected expressing genes in Poly(A) capture sequencing was slightly higher than that in Total RNA sequencing (Fig. 4A). Alternatively, the number in DNase I-treated sequencing was lower than that in the other two sequencings. However, about 90% of detected expressing genes were commonly detected (Fig. 4B). The expression pattern between these three sequencings showed a relatively high correlation (Fig. 4C). Also, differentially expressed genes (DEG) (FDR < 0.05) were only 90 genes, showing that the expression pattern was highly correlated (Fig. 4D). The hierarchical clustering analysis indicated that the expression pattern of DEG in Total RNA sequencing was more similar to that in DNase I-treated sequencing than that in Poly(A) capture sequencing, indicating that non-specific genomic DNA amplification affected quantification of expressed genes (Fig. 4D). The genes in cluster 1 were more highly expressed in Total RNA and DNase I-treated sequencings than in Poly(A) capture sequencing. The genes in cluster 2 were vice versa. Although the cluster 2 genes were almost coding genes, cluster 1 genes were almost non-coding genes that do not have poly(A) tail (Fig. 4E). Moreover, the cluster 1 genes contained histone genes, such as HIST1H3C, which also do not have poly(A) tail. It seems that cluster 1 genes represent non-specific genomic DNA amplification. Although cluster 2 genes contain non-coding genes, these genes exist in the intragenic regions of another genes, which may represent intron retention of mRNA of another genes. These results suggested that Poly(A) sequencing improves gene detection accuracy and expression patterns.

### Comparison between total RNA, DNase I-treated, and Poly(A) capture sequencing for alternative splicing analysis

**Figure 5:**
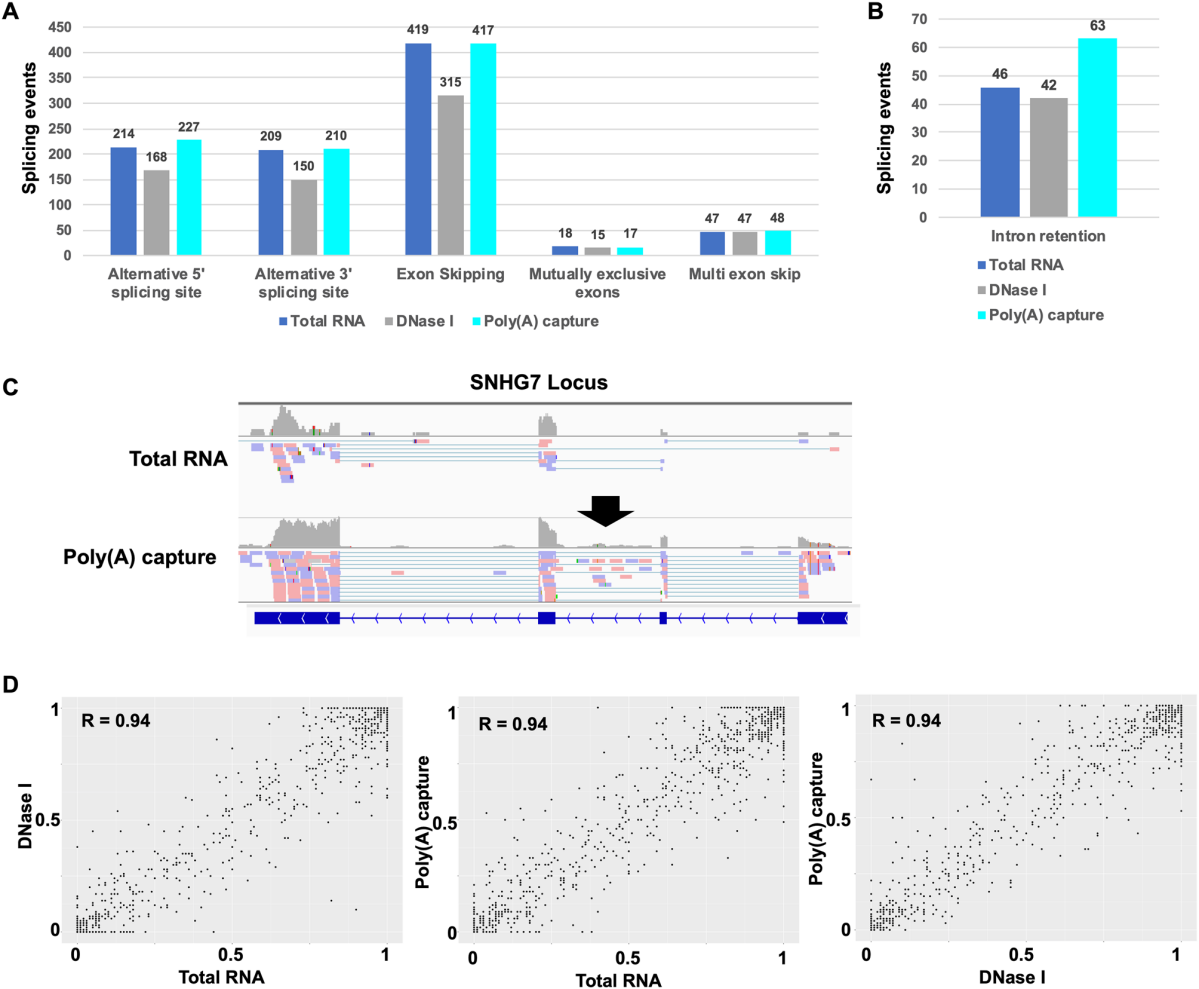
Comparison between Total RNA, DNase I-treated, and Poly(**A**) capture sequencings for alternative splicing analysis using SplAdder. The splicing event number per event patterns (alternative 5’ splicing site, alternative 3’ splicing site, Exon skipping, mutually exclusive exons, and multi-exon skip). (**B**) The number of intron retention. (**C**) The mapped reads at SNHG7 locus in the integrative genomic viewer (IGV). Black arrow indicated intron retention). (**D**) Scatter plot of the percent splicing index (PSI). The PSI of the splicing events were calculated by SplAdder. The *p*-value were calculated by t-test.

Next, for alternative splicing analysis, we used SplAdder software, which has been indicated to be superior to some other software such as rMATs, SpliceGrapher and JuncBase, to detect alternative splicing events^38^. To evaluate the performance of Total RNA, DNase I-treated, and Poly(A) capture sequencing, we compared the number and accuracy of the alternative splicing events between these three sequencings. The number of alternative 5’ splicing sites, alternative 3’ splicing sites, and Exon skipping in Poly(A) capture sequencing was slightly higher than that in Total RNA sequencing (Fig. 5A). Alternatively, the number in DNase I-treated sequencing was fewer than Total RNA sequencing. The number of mutually exclusive exons and multi-exon skip was almost the same between these three sequencings. The number of intron retention in Poly(A) capture sequencing was relatively more than the other two sequencings (Fig. 5B). The intron retention on the *SNHG7* gene was detected using only Poly(A) capture sequencing (Fig. 5C). The percentage spliced in the index (PSI) of the splicing event, which was commonly detected, highly correlated between these three sequencings, indicating that there is no significant difference in the alternative splicing events detected by these three methods as overall transcriptome (Fig. 5D). These results suggested that Poly(A) sequencing enhances the detection number and accuracy of minor alternative splicing events especially in the sense of detecting intron retention.

## Discussion

Fluctuations and regulation of mRNA splicing repertoire are associated with all aspects of biological activity. It will be more important to accurately analyze the state of the repertoire of mRNA splicing variants and its changes associated with the pathological condition to understand gene functions and life system. Next generation sequencing (NGS) is an analytical technology that can make this possible.

NGS-based applications for clinical laboratories also have been adopted as a gold standard for diagnostics of Mendelian-inherited diseases and cancers because of its analytic accuracy, high throughput, and cost-effectiveness. The VUS variants are half of all variants on Clinvar^17^, and its pathological significance should be determined. Presently, most NGS -based applications in clinical diagnosis target only coding regions, such as whole-exome and gene targeting panel sequencing. Nevertheless, the variants in the intron region, sometimes even in coding region, affect RNA splicing which causes protein damage. Although variants that may change RNA splicing can be computationally predicted, it is difficult to apply to clinical diagnosis because of an incomplete understanding of alternative splicing and normal transcriptome across tissues^4^. For this reason, in addition to the information of the traditional coding regions, it is necessary to determine whether an intron variant alters RNA splicing directly to confirm its pathogenicity in clinical situations, such as an analysis for undiagnosed inherited disease or cancer gene panel.

Current short-read sequencer-based transcriptome analysis cannot characterize the full-length transcripts because of the limitations of read length. Recently, long-read sequencers, such as Oxford Nanopore Technologies (ONT) and Pacific Biosciences (PacBio), have advanced and are gradually used because of their ability to overcome the limitations of read length^40–43^. Although the standard RNA-Seq method for short-read sequencers has been well-established, the standard RNA-Seq method is unavailable for long-read sequencers due to fragmentation at the library preparation step. On the other hand, the full-length double-strand cDNA sequencing method is available for long-read sequencing. Given these points, in order to clarify how DNA variants in deep intron or intragenic regions affect mRNA splicing, it seems useful to perform a DNA sequencing including non-coding regions and a full-length cDNA sequencing simultaneously with the same sample. Nevertheless, a previous study indicated that nonspecific genomic amplifications are yielded at the library preparation step and affect transcriptome analysis, such as transcript quantification and alternative splicing analysis^25^. This study also showed that the same results and non-specific genome amplification were yielded from only the genomic DNA. It is needed to eliminate contamination of genomic DNA for precise transcriptome analysis.

In this study, two methods were employed, which are the DNase I-treated full-length double-strand cDNA sequencing and poly(A) capture full-length double-strand cDNA sequencing to avoid non-specific genomic DNA amplification. Unexpectedly, non-specific genomic DNA amplification increased in the DNase I-treated sequencing than original Total RNA sequencing despite DNase I treatment. These non-specific genomic DNA amplifications affected quantification analysis and alternative splicing analysis. These non-specific genomic DNA amplifications might probably yield from the genome DNA that could not be completely digested in the DNase I-treated sequencing. It is possible that the efficiency of non-specific PCR amplification during double-strand cDNA synthesis in the SMARTer method was increased by digesting genomic DNA by DNase I and decreasing its molecular size. Alternatively, non-specific genomic DNA amplification was significantly reduced in poly(A) capture sequencing. The genes that are not actually detected, such as non-coding RNA, which does not have poly(A) tail, were detected in total RNA and DNase I-treated sequencings due to non-specific amplification noise. On the other hand, non-coding RNAs were undetected correctly in poly(A) capture sequencing. Complete novel splicing junctions were dramatically reduced in Poly(A) capture sequencing (Fig. 3E) comparing to the original Total RNA sequencing (Fig. 2D). That is, it was shown that many of the novel splicing variants detected in the total RNA sequence are artifacts. Moreover, alternative splicing events including intron retentions were more accurate and more prevalent in poly(A) capture sequencing.

Our poly(A) capture full-length double-strand cDNA sequencing improves the accuracy and detection ability of transcript quantification and alternative splicing events owing to the elimination of non-specific amplification noise from genomic DNA. Poly (A) capture sequences could be able to more accurately detect minor splicing events that occur in certain genes associated with cell differentiation or pathogenicity. Also, Poly(A) capture sequencing will provide information on all splice events in full-length transcripts because a highly accurate library created by this method can be applied to long-read sequencings, such as ONT and PacBio. Moreover, by combining with whole genome sequencing, this method will contribute to determine the VUS variants’ pathological significance that affects splicing events in clinical diagnosis.

## Supplementary Information

Supplementary Information 1.

Supplementary Information 2.

## Data Availability

The datasets have been deposited in the Gene Expression Omnibus (GEO) database. (GSE192928).
