# Supplementary material for: Poly(A) capture full length cDNA sequencing improves the accuracy and detection ability of transcript quantification and alternative splicing events
Source: Sci Rep. 2022 Jun 22;12:10599. doi: 10.1038/s41598-022-14902-7 (PMC9217819; doi:10.1038/s41598-022-14902-7)
Supplement: Supplementary file 1 — Supplementary Information 1. [file 41598_2022_14902_MOESM1_ESM.pdf]

Filename: 2021-03-26 - 12.39.56.RNA

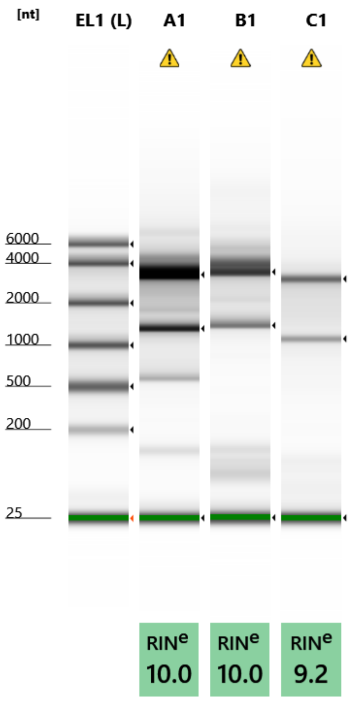

Default image (Contrast 100%)

Sample Info

Well	RINe	28S/18S (Area)	Conc. [ng/µl]	Sample Description	Alert	Observations
EL1	-	-	84.9	Electronic Ladder		Ladder
A1	10.0	3.0	162	iPS QIAGEN	⚠	Caution! Expired ScreenTape device
B1	10.0	1.6	82.8	iPS Trizol	⚠	Caution! Expired ScreenTape device
C1	9.2	2.0	46.6	iPS Trizol DnaseI	⚠	Caution! Expired ScreenTape device

 © Agilent Technologies, Inc. 2019

Peak: EL1: Electronic Ladder

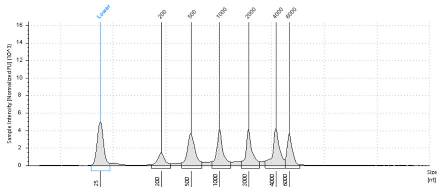

**Location**	EL1
**RINe**	-
**UpperSLowerSArea**	-
**Concentration**	84.9
**Description**	Electronic Ladder
**Observations**	Ladder

Size [nt]	Calibrated Conc. [ng/µl]	Assigned Conc. [ng/µl]	Peak Molarity [nmol/l]	% Integrated Area	Peak Comment	Observations
25	40.0	40.0	4710	-		Lower Marker
200	5.94	-	87.4	7.80		
500	15.9	-	93.6	20.88		
1000	14.2	-	41.8	18.62		
2000	13.8	-	20.3	18.11		
4000	15.5	-	11.4	20.38		
6000	10.8	-	5.31	14.22		

Peak: A1: iPS QIAGEN

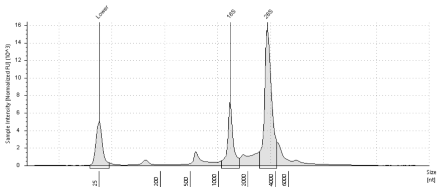

**Location**	A1
**RINe**	10.0
**UpperSLowerSArea**	3.0
**Concentration**	162
**Description**	iPS QIAGEN
**Alert**	⚠
**Observations**	Caution! Expired ScreenTape device

Size [nt]	Calibrated Conc. [ng/µl]	Assigned Conc. [ng/µl]	Peak Molarity [nmol/l]	% Integrated Area	Peak Comment	Observations
25	40.0	40.0	4710	-		Lower Marker
1307	27.5	-	62.0	25.27		18S
3264	81.4	-	73.4	74.73		28S

Peak: B1: iPS Trizol

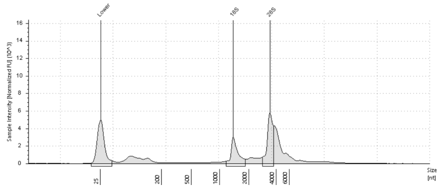

**Location**	B1
**RINe**	10.0
**UpperSLowerSArea**	1.6
**Concentration**	82.8
**Description**	iPS Trizol
**Alert**	⚠
**Observations**	Caution! Expired ScreenTape device

Size [nt]	Calibrated Conc. [ng/µl]	Assigned Conc. [ng/µl]	Peak Molarity [nmol/l]	% Integrated Area	Peak Comment	Observations
25	40.0	40.0	4710	-		Lower Marker
1376	13.3	-	28.5	38.61		18S
3434	21.2	-	18.1	61.39		28S

Peak: C1: iPS Trizol DnaseI

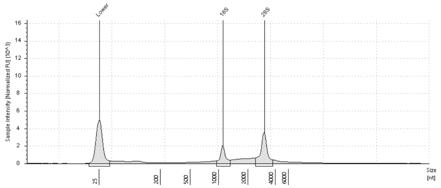

**Location**	C1
**RINe**	9.2
**UpperSLowerSArea**	2.0
**Concentration**	46.6
**Description**	iPS Trizol DnaseI
**Alert**	⚠
**Observations**	Caution! Expired ScreenTape device

Size [nt]	Calibrated Conc. [ng/µl]	Assigned Conc. [ng/µl]	Peak Molarity [nmol/l]	% Integrated Area	Peak Comment	Observations
25	40.0	40.0	4710	-		Lower Marker
1108	7.56	-	20.1	33.40		18S
3043	15.1	-	14.6	66.60		28S

© Agilent Technologies, Inc. 2019

© Agilent Technologies, Inc. 2019
